# Aortic arch shape is not associated with hypertensive response to exercise in patients with repaired congenital heart diseases

**DOI:** 10.1186/1532-429X-15-101

**Published:** 2013-11-12

**Authors:** Hopewell N Ntsinjana, Giovanni Biglino, Claudio Capelli, Oliver Tann, Alessandro Giardini, Graham Derrick, Silvia Schievano, Andrew M Taylor

**Affiliations:** 1 Centre for Cardiovascular Imaging, UCL Institute of Cardiovascular Science & Cardiorespiratory Unit, Great Ormond Street Hospital for Children, NHS Trust, London, UK; 2 Cardiorespiratory Unit, Level 7, Nurses Home, Great Ormond Street Hospital for Children, Great Ormond Street, London WC1N 3JH, UK

**Keywords:** Coarctation, Arterial switch operation, Anatomical models, Blood pressure, Exercise test

## Abstract

**Background:**

Aortic arch geometry is linked to abnormal blood pressure (BP) response to maximum exercise. This study aims to quantitatively assess whether aortic arch geometry plays a role in blood pressure (BP) response to exercise.

**Methods:**

60 age- and BSA-matched subjects – 20 post-aortic coarctation (CoA) repair, 20 transposition of great arteries post arterial switch operation (ASO) and 20 healthy controls – had a three-dimensional (3D), whole heart magnetic resonance angiography (MRA) at 1.5 Tesla, 3D geometric reconstructions created from the MRA. All subjects underwent cardiopulmonary exercise test on the same day as MRA using an ergometer cycle with manual BP measurements. Geometric analysis and their correlation with BP at peak exercise were assessed.

**Results:**

Arch curvature was similarly acute in both the post-CoA and ASO cases [0.05 ± 0.01 vs. 0.05 ± 0.01 (1/mm/m^2^); p = 1.0] and significantly different to that of normal healthy controls [0.05 ± 0.01 vs. 0.03 ± 0.01 (1/mm/m^2^), p < 0.001]. Indexed transverse arch cross sectional area were significantly abnormal in the post-CoA cases compared to the ASO cases (117.8 ± 47.7 vs. 221.3 ± 44.6; p < 0.001) and controls (117.8 ± 47.7 vs. 157.5 ± 27.2 mm^2^; p = 0.003). BP response to peak exercise did not correlate with arch curvature (r = 0.203, p = 0.120), but showed inverse correlation with indexed minimum cross sectional area of transverse arch and isthmus (r = -0.364, p = 0.004), and ratios of minimum arch area/ descending diameter (r = -0.491, p < 0.001).

**Conclusion:**

Transverse arch and isthmus hypoplasia, rather than acute arch angulation plays a role in the pathophysiology of BP response to peak exercise following CoA repair.

## Background

Both exercise induced and resting arterial hypertension following a successful repair of the coarctation of the aorta (CoA) are well-recognised complications [1-4]. In the general population, exercise induced hypertension has been linked to an increased risk of developing systemic hypertension during early adulthood [5,6]. It has been suggested that as part of post surgical follow-up of CoA patients, exercise testing would help with the identification of re-coarctation and of abnormal blood pressure (BP) response [7]. Amongst other suggested aetiologies for residual hypertension [8] is the 'gothic’ morphology that the arch can assume following CoA repair irrespective of type of surgery [9,10]. Previously described as a triangular shaped arch, the 'gothic’ deformity of the aortic arch is also known to occur following arterial switch operation (ASO) for transposition of great arteries (TGA) [11]. In TGA post-ASO subjects, this occurs as a result of the 'Lecompte manoeuvre’, which positions the main pulmonary artery anterior to the aorta thus pushing the aorta posteriorly [12]. However, there are no reported data on abnormal BP response to exercise in ASO patients, despite the similarity in aortic arch morphology with post-CoA repair patients [13,14].

This study aims to quantify aortic arch geometry in 3D and test the hypothesis that “gothic” arch morphology in its own right is not associated with abnormal BP response during exercise.

## Methods

### Patients

We studied 20 patients with repaired CoA, whose aortic arches were described as 'gothic’ based on cardiovascular magnetic resonance (CMR), and 20 TGA patients who had undergone ASO. These patient groups were compared with 20 age- and BSA-matched control subjects who had undergone CMR imaging as part of screening for familial cardiomyopathy and reported as normal on cardiac assessment. All patients were asymptomatic and were not on anti-hypertensive medication. Data acquisitions were approved by the local institutional ethics committee of Great Ormond Street Hospital for Children NHS Foundation Trust, and all patients and/or their parents/guardians gave informed consent for use of their data.

### Cardiopulmonary exercise test

All subjects had a cardiopulmonary exercise test (CPET) on the same day as CMR imaging. The exercise test was performed using an electronically braked ergometer cycle with respiratory gas analysis. The ramp protocol was used, with a 3-minute load-less cycling followed by work rate increments of 10 to 20 W/minute aiming to achieve the maximum expected workload in 10 to 12 minutes of exercise. The maximum workload was based on subject’s weight (Kg) (3 W/Kg for females and 3.5 W/Kg for males). A 12-lead electrocardiogram and transcutaneous oxygen saturation were also monitored throughout the study. Blood pressure was measured at rest using an appropriately sized cuff with a mercury sphygmomanometer, and every 2 minutes thereafter during exercise in the right arm.

### CMR data

All subjects underwent CMR examination using a 1.5 T Avanto MR scanner (Siemens Medical Solutions, Erlangen, Germany). A 3D balanced, steady-state free precession (bSSFP) whole heart, free breathing isotropic data acquisition method was used to obtain a 3D volume of the left ventricle and aorta during mid-diastolic rest period (sequence parameters: TR 3.0, TE 1.5, flip angle 90°, number of lines per segment acquired per cardiac cycle 30–40, sensitivity-encoding factor 2.0, bandwidth per pixel 590 Hz, field of view 280 × 280 × 120 mm, acquisition matrix 192 × 192 × 80 and iso-volumetric voxel size 1.5 × 1.5 × 1.5 mm). Magnetisation preparation was achieved by applying a T2-weighted preparation pulse with an echo time of 50 ms and a frequency-selective fat-saturation pulse followed by a spoiler gradient. A customized shim procedure was applied to the volume of the entire heart. For real-time respiratory gating and real-time correction of the 3D volume in the cranio-caudal direction, a navigator echo was acquired from a cylindrical region that was generated by a two-dimensional excitation pulse perpendicular to the right hemi-diaphragm with a gating window of ±3 mm.

Distensibility was calculated from CMR data using the formula:

$$\mathrm{Dist}\;\left({\mathrm{mmHg}}^{-1}\right)=\left[\left(A_{\max}–A_{\min}\right)/\left(A_{\min}x\;\left(P_{\max}-P_{\min}\right)\right)\right]$$

Where A_max_ and A_min_ are cross-sectional areas of the aortic root in systole and diastole respectively, and P_max_and P_min_ are systolic and diastolic blood pressures [15].

### Geometric reconstruction and analysis

Whole-heart images were used for anatomical reconstructions using commercial software (Mimics v15, Materialise, Leuven, Belgium) according to previously described and validated methodologies [16]. CMR data were segmented and a 3D volume of the heart and vessels was obtained by means of operations of thresholding and region-growing. The original rough 3D volume was cropped such that only the following anatomical elements of interest were kept: the aortic root, the aortic arch, and the descending aorta up to the level of the diaphragm. With regard to the brachiocephalic vessels, their origins from the aortic arch were kept as an anatomical landmark. The resulting 3D volumes were then smoothed (smoothing parameters: smooth factor = 0.5, iterations = 2) and the centreline calculated for the aortic vessel from the centre point of the aortic valve to the level of the diaphragm.

**Figure 1:**
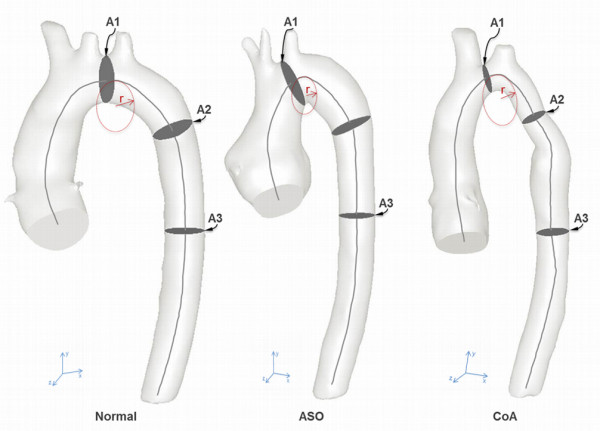
**Patient-specific 3D geometric reconstructions of aortas.** Normal (healthy subject), ASO (arterial switch operated subject), CoA (coarctation of the aorta subject) displaying geometric landmarks of centreline from the centre point of the aortic valve to the descending aorta at the level of the diaphragm, circle tangent to the highest point of the vessel centreline with the radius (r) for assessment of arch curvature, and positions of measurements for cross sectional areasA1 (transverse arch), A2 (isthmus) and A3 (descending aorta) as labelled.

The following anatomical parameters were quantified for each subject (Figure 1):

a. Aortic arch curvature quantified as follows: firstly, the vessel centreline was extracted from the 3D geometry of the aorta of each subject from the coaption point to the level of diaphragm. Secondly, this centreline was imported into software for computer-assisted design (Rhinoceros® 5, Robert, McNeel and Associates, Seattle, WA, USA) for curvature analysis. Analysis of the curvature was performed using the curvature circle tool, which allows visualization of the tangent osculating circle. This osculating circle was tracked along the 3D trajectory of the centreline until it reached its minimum size in correspondence to the highest point of the arch. Finally, the curvature of the centreline in such position was calculated as the inverse of radius (1/r) of the osculating circle as per mathematical definition [17,18]. Also the aortic arch curvature was indexed over BSA.

b. Aortic cross sectional areas planimeterized orthogonal to the central line, namely the transverse arch (A1), the aortic isthmus (in the presence of CoA) or equivalent (A2), and the descending aorta (A3).

### Statistical analysis

SPSS version 21 (SPSS Inc., Chicago, IL, USA) was used for all analyses. Data are expressed as mean ± standard deviation. A two-sided p-value of 0.05 or less and 95% confidence intervals were used to report the statistical significance and the precision of our results, respectively. Between-groups comparison of continuous variables (control vs. ASO vs. CoA subjects) was performed using one-way analysis of variance (one-way ANOVA) followed by post-hoc analysis for parametric variables. Pearson’s coefficient of correlation was used to assess the degree of correlation between systolic BP at peak exercise and the following:

•Indexed aortic arch curvature,

•Indexed minimum transverse and isthmic cross sectional areas, and

•Ratios of minimum transverse and isthmic cross sectional areas to descending aortic area.

Using peak systolic BP as a dependent variable, linear regression analysis was used to assess for independent predictors of BP response during exercise amongst the co-variants.

## Results

### Patient characteristics and baseline measurements

Subject characteristics, including baseline measurements of BP, heart rate and anthropometries are reported in Table 1. All study subjects were matched for age in years – 15.2 ± 2.0 for controls, 15.0 ± 2.1 for ASO, and 16.5 ± 3.1 for CoA cases (p = 0.123) – and body surface area – 1.75 ± 0.25 m^2^ for controls, 1.58 ± 0.26 m^2^ for ASO, and 1.67 ± 0.26 m^2^ for CoA cases (p = 0.121). All subjects had left sided aortic arch with normal branching pattern of the head and neck vessels unless otherwise stated to have had a left subclavian patch repair for the Coarctation cohort (Table 2). No significant difference in baseline heart rate and diastolic BP between the three groups was identified. Systolic BP at rest was significantly higher in CoA patients compared to healthy controls (123 ± 11 mmHg, Vs. 111 ± 16 mmHg, p = 0.005) and ASO subjects (123 ± 11, Vs. 110 ± 13 mmHg, p = 0.006).

**Table 1:** Patient characteristics and baseline measurements

***Variables***	**Control**	**ASO**	**CoA**
N	20	20	20
Sex (m/f)	17/3	16/4	13/7
Ages (Years)	15.2 ± 2.0	15.0 ± 2.1	16.5 ± 3.1
Height (cm)	169.9 ± 13.0	164.4 ± 13.9	167.2 ± 13.5
BSA (mm^2^)	1.75 ± 0.25	1.58 ± 0.26	1.67 ± 0.26
Heart rate at rest (bpm)	93 ± 15	81 ± 11	91 ± 14
Diastolic BP at rest (mmHg)	65 ± 11	65 ± 10	71 ± 10
Systolic BP at rest (mmHg)	111 ± 16	111 ± 13	123 ± 11

BP (blood pressure), m (Male), f (Female).

**Table 2:** Details of the coarctation patients

**Case no.**	**Sex**	**Age at CMR and CPEX**	**BSA (m**^**2**^**)**	**Age at coarctectomy**	**CPB**	**Type of surgery**	**BSBP (mmHg)**	**PSBP on exercise (mmHg)**
1	M	17.3	2.00	4 m	No	E-E	132	218
2	M	18.3	1.70	6y	No	SCF	136	196
3	M	11.1	1.10	2 m	No	E-E	132	152
4	M	16.6	1.70	3 m	Yes	Ext E-E	116	155
5	F	13.7	1.88	5y	No	P-A	138	212
6	F	13.3	1.55	6d	No	P-A	120	168
7	M	20.1	1.90	5y	No	E-E	140	230
8	F	17.3	1.65	6 m	No	E-E	92	160
9	F	16.5	1.50	4 m	No	E-E	120	170
10	M	11.9	1.33	3 m	No	E-E	120	145
11	M	18.3	1.80	4d	No	E-E	120	230
12	M	10.3	1.28	9d	No	SCF	132	220
13	F	21.9	1.90	1 m	No	E-E	125	210
14	F	16.4	1.25	7 m	No	E-E	124	158
15	M	17.4	1.75	10d	No	E-E	118	150
16	F	18.4	1.90	4 m	Yes	Ext E-E	102	134
17	M	18.0	2.02	2y	No	E-E	128	174
18	M	15.8	1.74	3 m	Yes	E-E	125	164
19	M	16.9	1.77	9d	No	E-E	125	210
20	M	20.1	1.70	6d	No	E-E	120	144

M (male), F (Female), d (days), m (months), y (year), CPB (cardiopulmonary bypass), E-E (End to end anastomosis), SCF (Subclavian flap), Ext E-E (Extended end to end anastomosis, P-A (Patch aortoplasty), BSBP (Baseline systolic blood pressure), PSBP (Peak systolic blood pressure).

**Table 3:** Peak exercise and geometrical measurements and aortic distensibility of the 3 study groups

***Variables***				***P values***		
	**Control**	**ASO**	**CoA**	**Control/**	**Control/**	**ASO/**
	**(n = 20)**	**(n = 20)**	**(n = 20)**	**ASO**	**CoA**	**CoA**
VO2 Max (ml/Kg/min )	38.9 ± 8.3	37.3 ± 8.8	33.9 ± 8.0	0.532	0.063	0.210
Peak heart rate (bpm)	180 ± 17	174 ± 16	168 ± 22	0.323	0.044	0.295
Peak diastolic BP (mmHg)	64 ± 9	64 ± 10	69 ± 11	0.891	0.135	0.173
Peak systolic BP (mmHg)	153 ± 20	146 ± 20	180 ± 32	0.360	0.001*	<0.001*
iCurvature [(1/mm)/m^2^]	0.03 ± 0.01	0.05 ± 0.01	0.05 ± 0.01	<0.001*	<0.001*	1.000
Curvature [(1/mm]	0.05 ± 0.01	0.07 ± 0.01	0.08 ± 0.03	<0.001*	<0.001*	0.277
iTransverse arch (mm^2^/m^2^)	157.5 ± 27.2	221.3 ± 44.6	117.8 ± 47.7	<0.001*	0.003*	<0.001*
iIsthmus (mm^2^/m^2^)	142.5 ± 33.7	186.4 ± 25.9	133.0 ± 68.6	<0.001*	0.721	<0.001*
Descending aortic (mm^2^/m^2^)	115.7 ± 22.6	136.6 ± 24.2	143.5 ± 49.0	0.058	0.038^*^	0.525
Transverse/descending ratio	1.29 ± 0.78	1.40 ± 0.10	0.98 ± 0.23	0.177	<0.001*	<0.001*
Isthmus/descending ratio	1.09 ± 0.52	1.20 ± 0.11	0.97 ± 0.19	0.001*	0.006*	<0.001*
Distensibility (×10^-3^ 1/mmHg)	6.7 ± 3.7	2.1 ± 1.9	3.6 ± 3.2	<0.001*	0.003*	1.220

iCurvature (indexed curvature), iIsthimus (Indexed Isthmus cross sectional area), iTransverse (Indexed Transverse cross sectional area) BP (blood pressure), Values expressed as mean ± standard deviation, *indicates significant p value.

### Aortic arch morphology

**Figure 2:**
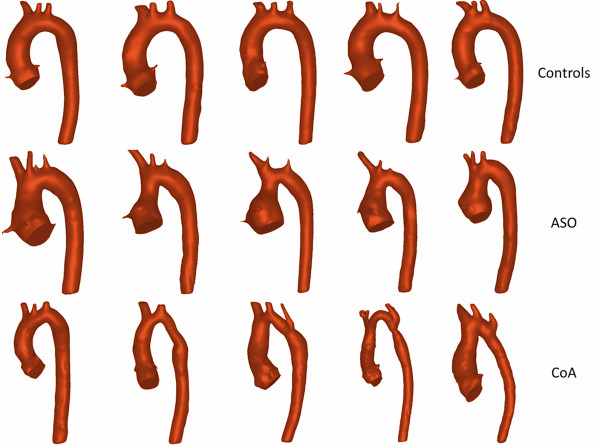
**Five examples for each group of 3D anatomical reconstructions.** 5 cases healthy controls (top panel) with smooth arch curvature, 5 ASO cases(middle panel) with acute arch angulation and 5 cases of CoA post repair (bottom panel), with acute angulation and some hypoplastic transverse arch and isthmus).

Aortic arch geometry measurements are reported in Table 3, and examples of 3D reconstructions of healthy controls, ASO and CoA cases are shown in Figure 2. The aortic arch curvature of CoA subjects previously defined as 'gothic’ on bSSFP 3D CMR was significantly more acute compared to controls (p < 0.001). ASO subjects had a significantly acute arch curvature with respect to controls (p < 0.001) but no difference with CoA individuals (p = 1.0). The transverse arch cross sectional area was significantly smaller in CoA patients compared to both controls (p < 0.001) and ASO subjects (p = 0.003). The ratio of transverse arch to descending aortic area was significantly smaller in CoA patients compared to both controls (p < 0.001) and ASO subject (p < 0.001).

### Blood pressure response to exercise and correlation with arch geometry

**Figure 3:**
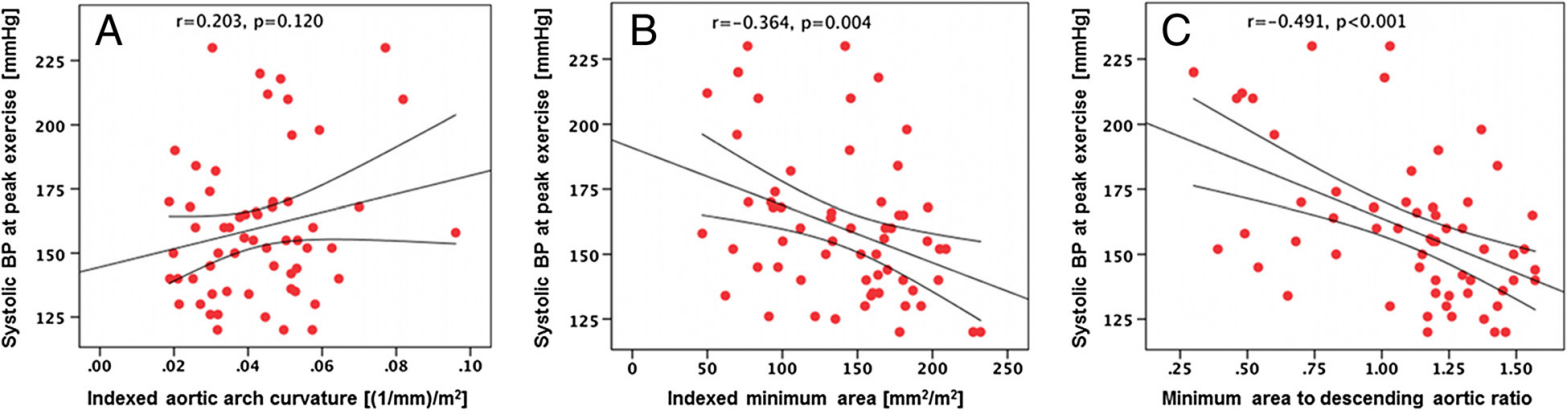
**Scatter plots displaying correlations of systolic BP at peak exercise with various geometric measures. (A)** BSA indexed aortic arch curvature, **(B)** indexed minimum aortic arch area at the level of transverse segment or isthmus, and **(C)** Ratio of the minimum area at the level of transverse arch or isthmus to descending aortic ratio respectively. Letter (r) denotes correlation coefficient with an accompanying sign of dispersion –p values are reported for statistical significance at p = 0.05.

The BP measurements acquired during CPET and distensibility from CMR data are shown in Table 3. Peak exercise systolic BP was significantly higher in CoA patients compared to both controls (180 ± 32 mmHg, Vs. 153 ± 20 mmHg, p =0.001) and ASO cases (180 ± 32 mmHg, Vs. 146 ± 20 mmHg, p < 0.001) respectively, with no significant difference between controls and ASO cases (153 ± 20 mmHg, Vs. 146 ± 20 mmHg, p = 0.36). Both coarctation post-repair and ASO patients were less distensible compared to normal healthy controls (Table 3). The results of correlations between arch geometry and systolic BP at peak exercise are shown in Figure 3. There was no significant correlation observed between aortic arch curvature and systolic BP response to peak exercise. A significant negative correlation was observed between systolic BP at peak exercise and minimal cross sectional areas at the transverse arch and isthmus. The ratio of the minimal cross sectional area to descending aortic area showed a significant negative correlation with systolic BP at peak exercise.

Multiple linear regression analysis showed that only base line BP was a significant independent predictor of systolic BP at peak exercise, with (standardised beta coefficient =0.473 and p < 0.001).

## Discussion

This study has assessed the influence of aortic arch geometry on BP response to exercise in CoA post-repair, TGA post-ASO and healthy control subjects. To our knowledge, this is the first study that has combined ASO subjects and CoA repaired cohorts, based on their similar 'gothic’ aortic arch morphology.

Following repair of CoA, patients are at risk of developing sustained arterial hypertension in later life, particularly if they have an abnormal BP response to exercise [5,19]. Abnormal aortic arch geometry, mainly 'gothic’ arch morphology, has been reported to be associated with a higher prevalence of hypertensive BP response to exercise in patients who have undergone surgical repair of aorta coarctation [10].

In our study, we have shown that using a quantitative, 3D assessment of arch curvature patients with repaired CoA and TGA repaired with ASO have similar, acutely angulated 'gothic’ aortic arch compared to healthy control individuals. This would suggest similar behaviour in terms of blood flow profiles across this segment. Interestingly, Ou et al have shown that the 'gothic’ arch geometry is associated with resting hypertension [10] in one study, and abnormal BP response to exercise of otherwise normotensive subjects in another study of patients with successfully repaired CoA [10,20]. Our data agree with these findings, showing significantly higher resting systolic BP and high systolic BP at peak exercise in CoA subjects with 'gothic’ shaped arch, compared to TGA post ASO and healthy control subjects. However, our findings do not show good correlations between abnormal BP response to exercise and geometric measures of aortic arch curvature. Indeed, despite very similar, abnormal aortic arch shapes between our two patient groups, it is only those with repaired CoA that have an abnormal, hypertensive BP response to exercise.

This begs the question – What is the reason for the different BP responses to exercise between these 2 patient groups? In our cohort, patients post-CoA repair did have smaller cross sectional areas of the transverse arch and isthmus, and when the minimal transverse arch/isthmus area was correlated with the BP response at exercise, there was a negative correlation. Thus residual narrowing accounts for a significant amount of the hypertensive BP response seen in our post-CoA repair patients, albeit it contributing to less than 50% of influence into BP change during maximal exercise suggesting that there are other factors potentially contributing to abnormal BP response that are not accounted for in this study. These factors could be: the material properties in the aortic wall; altered responses of renin-angiotensin system; abnormal baroreceptor function; and abnormal vascular bed of the pre-coarctation segment [8,21-23].

In this study both ASO and post CoA repair patients have significantly reduced ascending aortic distensibility in keeping with literature [23-25], but interestingly only the post CoA repair subjects have reduced exercise capacity compared to controls on average. This would suggest that it is the abnormal vascular activity rather than conduit stiffness alone that affects exercise response in these patients.

From a methodological perspective, geometrical analysis performed on 3D volumes derived from imaging data allows more accurate and comprehensive assessment of the anatomy. It enables, in fact, to better evaluate the spatial arrangement of the vessel and to take into account the possible out of plane positioning of the aortic centreline, with respect to a 2D visual assessment. Dimensions, such as aortic cross sectional areas at various locations, can also be more confidently estimated from 3D models, rather than a line adjoining the vessel walls. Moreover, shape and vessel tortuosity can only be appreciated in 3D. In this study, for instance, qualitative assessment based on the 3D models highlighted that CoA patients display a more varied morphology, with some subjects presenting more tortuous arches than others, whereas ASO and healthy geometries are more uniform (Figure 2). Finally, in the future these models could be employed in computational fluid dynamics or fluid–structure interaction simulations, combining in-silico (i.e. computational) hemodynamic results with clinical data at a patient-specific level to identify and characterize the other parameters involved in abnormal BP response in these patients.

## Conclusions

This study shows that arch morphology, and specifically 'gothic’ aortic arch shape, as derived from 3D models does not impact on abnormal BP response, as demonstrated by the comparison between data from patients with repaired CoA and patients with surgically repaired TGA. Residual aortic arch hypoplasia and isthmus narrowing were identified as parameters affecting the pathophysiology of BP response to exercise. Our results therefore, suggest that both surgeons and interventionists should pay particular attention to the size of the residual arch when they manage these patients.

## Competing interest

AMT has a research agreement with Siemens Medical Solutions. All authors declare no other relationships or activities that could appear to have influenced the submitted work. Other authors declare no competing interest.

## Authors’ contributions

HNN, GB, CC, SS, and AMT designed the study, contributed to the data acquisition, analysis, and data interpretation. They drafted the manuscript. HNN, GB, CC, OT, AG, GD, SS, and AMT contributed to the data acquisition, analysis, interpretation of results, and revised critically the manuscript. HNN, GB, CC, SS, and AMT contributed to data collection, data interpretation, and made a critical revision of the manuscript. All authors read and approved the final manuscript.
